# Comparing GreenLight PVP and HoLEP beyond 5 years: A systematic review of long‐term functional outcomes and reoperation rates

**DOI:** 10.1002/bco2.483

**Published:** 2025-02-17

**Authors:** Arthur Yim, Matthew Alberto, Xingqi Yan, Damien Bolton, Lih‐Ming Wong, Kapil Sethi

**Affiliations:** ^1^ Department of Urology Austin Health Heidelberg Victoria Australia; ^2^ Department of Surgery The University of Melbourne Melbourne Victoria Australia; ^3^ Young Urology Researchers Organisation (YURO) Melbourne Victoria Australia

**Keywords:** benign prostatic hyperplasia, durability, functional outcomes, GreenLight photoselective vaporisation of prostate, holmium laser enucleation of prostate, reoperation

## Abstract

**Objectives:**

This study aimed to compare long‐term (≥ 5 years) functional outcomes and reoperation rates following holmium laser enucleation of prostate (HoLEP) vs GreenLight photoselective vaporisation of prostate (GLPVP).

**Methods:**

MEDLINE, Embase and Cochrane databases were searched from inception to December 2023. Included were randomised controlled trials (RCTs), cohort studies and case series studying HoLEP and/or GLPVP, where functional outcomes and reoperation rates were reported. Studies with <5‐year follow‐up were excluded. Evidence was synthesised as a comparison across all parameters. Quality of evidence was assessed with the Newcastle–Ottawa Scale.

**Results:**

Of 3047 records identified, 25 were eligible, including two RCTs, two cohort studies, one cross‐sectional study and 20 case series. Twenty‐three studies focused on HoLEP or GLPVP, whilst two were comparative studies. HoLEP demonstrated long‐term durability of outcomes and low reoperation rates (mean 4.1%, range 2.0%–6.3%) at a mean follow‐up of 7.3 years. GLPVP also had durable outcomes at 5‐year follow‐up, but inconclusive evidence for improvements at 10 years. Reoperation rates were also higher (mean 12.6%, range 3.8%–33.3%). This is in keeping with findings of comparative studies, where HoLEP demonstrated greater improvements in all functional parameters except PVR, and lower reoperation rates. Findings are limited by patient attrition, lack of comparative studies and long‐term data beyond 10 years. Three studies examined the 180‐W GLPVP model at 5 years showed superior durability to earlier 80‐W/120‐W models.

**Conclusions:**

Current evidence suggests that HoLEP provides significantly greater functional improvements and a lower reoperation rate when compared with the GLPVP 80‐W/120‐W model at 5‐year follow‐up. The 180‐W model is comparable with HoLEP based on limited data at 5 years, but there is a lack of data beyond 10 years for longer‐term functional outcomes.

## 1 INTRODUCTION

Benign prostatic hyperplasia (BPH) is recognised as one of the major causes of lower urinary tract symptoms (LUTS) in elderly men. Patients with LUTS that are not responsive to medical therapy often require surgical treatment. Transurethral resection of the prostate (TURP) has been long recognised as the gold standard for treatment of LUTS secondary to BPH with prostate volumes smaller than 80–100 mL, while open prostatectomy was historically considered the treatment of choice for larger sized prostates before HoLEP.^1^ However, despite the excellent efficacy and durability of TURP and open prostatectomy, they are associated with significant morbidity.^2^

Over the past two decades, the introduction of laser energy in treatment of BPH has drastically changed the landscape for BPH surgery. Two notable candidates among laser treatments are holmium laser enucleation of prostate (HoLEP) and GreenLight photoselective vaporisation of prostate (GLPVP), both demonstrating excellent safety and efficacy profile in the short‐term non‐inferior to TURP, and consequently are gradually being accepted as valid alternatives.^3^, ^4^ An overview comparing the two techniques is provided in Table 1.

**TABLE 1:** Overview and comparison of GreenLight photoselective vaporisation of prostate (GLPVP) and holmium laser enucleation of the prostate (HoLEP).

	GreenLight photoselective vaporisation of prostate (GLPVP)	Holmium laser enucleation of the prostate (HoLEP)
Purpose	Relieve symptoms of BPH by reducing prostate tissue	Treat BPH by removing excess prostate tissue
Laser type	Green laser (532 nm wavelength)	Holmium laser (2100 nm wavelength)
Procedure/technique	Photoselective vaporisation of the prostate	Enucleation and morcellation of prostate tissue
Tissue ablation	Vaporisation of excess prostate tissue	Enucleation (removal) of adenoma tissue
Coagulation	Provides haemostasis through coagulation	Simultaneous cutting and coagulation for haemostasis
Hospital stay	Often outpatient or short hospital stay	Usually requires an overnight hospital stay
Prostate size limit	Effective for moderate‐sized prostates	Effective for larger prostates
Postoperative complications	Minimal, but can include irritative voiding symptoms and haematuria	Minimal, but can include retrograde ejaculation and urinary incontinence
Sexual function	Preserves sexual function in most cases	May cause retrograde ejaculation in some cases
Recovery time	Rapid recovery and return to normal activities	Recovery may take a bit longer

HoLEP technique was developed in the 1990s and has been described numerous times previously.^5^ It utilises a 2140‐nm pulsed mode laser generated from the holmium:yttrium aluminium garnet (Ho:YAG) crystal (100 W/120 W platform, Lumenis, Yokneam, Israel). A front‐firing 550‐μm fibre is commonly set to a frequency of 50 Hz and energy of 2.0 J, whilst haemostasis settings are 1.5 J/30 Hz with a wide pulse. HoLEP is conceptually the endoscopic approach of an open simple prostatectomy and utilises a 26Fr continuous flow endoscope with a laser bridge. Morcellation requires a nephroscope. Traditional laser enucleation requires release of the three prostatic lobes (median and two lateral) into the bladder. Haemostasis is optimised by defocusing the laser fibre tip away from bleeding tissue which causes blanching. Furthermore, enucleation using HoLEP also causes haemostasis due to the unique property of the holmium laser to coagulate whilst cutting tissue. Depth of tissue penetration is 0.4 mm with effective coagulation of up to 3 mm. More contemporary techniques have also been utilised, including en bloc enucleation and the two‐lobe enucleation technique.^5^

Alternatively, GLPVP utilises a GreenLight laser (American Medical Systems, Inc., Minnetonka, USA) with a 523‐nm laser and is selectively absorbed by haemoglobin thereby resulting in effective vaporisation of well‐vascularised prostatic tissue.^6^ Multiple systems have been developed from 80 W potassium titanyl phosphate, high‐pressure sodium 120‐W lithium triborate and the most recent Xcelerated Performance System (XPS) 180 W lithium triborate system in 2010. Similar to HoLEP, a 26Fr continuous flow endoscope is utilised; however, a sweeping motion is employed via a side‐firing laser to an optimal of 30 degrees with the aim of heating tissue to 100°C for effective vaporisation. The key indicator of efficient vaporisation is the production of bubbles and again focusing on treating the median and lateral lobes.^6^

Two major considerations in adoption of these contemporary techniques are the long‐term durability of improvements in functional outcomes and reoperation rates. Literature published over the past two decades have accumulated extensive evidence on the safety and efficacy of HoLEP and GLPVP in the short‐term and intermediate term; however, comparison of long‐term functional outcomes and reoperation rates is lacking. This is paramount to development of evidence‐based recommendations and for widespread implementation.

The aim of this paper is to compare these two modalities of surgical treatment for BPH by assessing the durability of long‐term improvements in functional outcomes, adverse events and reoperation rates.

## 2 METHODS

This review was performed in accordance with the PRISMA 2020 statement.^7^ It was registered with the International Prospective Register of Systematic Reviews (PROSPERO #CRD42023453125), where the protocol and search strategy are available.

### 2.1 Eligibility criteria

A summary of eligibility criteria for this review, following the PICO framework (Population, Intervention, Comparison, Outcome) is detailed in Table 2. Articles were included if they were randomised controlled trials (RCTs), cohort studies or case series aiming to study long‐term (minimum 5 years) functional outcomes and reoperation rates of either GLPVP, HoLEP or both. Studies were included if they used at least one standardised functional parameter, including international prostate symptom score (IPSS), quality of life (QoL) index, peak flow rate (Qmax) and post‐void residual volume (PVR), or if they provided data on reoperation due to any causes throughout the follow‐up period.

**TABLE 2:** Criteria for studies included in this systematic review.

Inclusion criteria	
Population	Male patients with LUTS suitable for surgical management
Intervention	HoLEP
Comparison	GLPVP
Outcomes	Minimum of 5‐year follow‐up with IPSS or QoL index or Qmax or PVR or reoperation rate
Setting	RCT, cohort study or case series

Abbreviations: GLPVP, GreenLight photoselective vaporisation of prostate; HoLEP, holmium laser enucleation of prostate; IPSS, international prostate symptom score; LUTS, Lower urinary tract symptoms; RCT, randomised controlled trial; QoL: quality of life.

Articles were excluded if the study focused on diseases other than BPH, or if BPH was complicated by confounding factors such as previous urological or prostatic surgeries and comorbidities that could affect functional outcomes after PVP or HoLEP treatment, including development of prostatic cancer, neurogenic bladder and neurological diseases including Parkinson's disease and stroke. Studies were also excluded if no patients in the study completed a minimum of 5‐year follow‐up. Studies for which data are not provided for any of the relevant parameters, or with full‐text unavailable, were also excluded. Only peer‐reviewed articles in English published within the past 20 years were considered.

### 2.2 Search strategy

An online electronic database search was undertaken using the platforms of MEDLINE, Embase and Cochrane Library on 1 November 2023. Initial search utilised MeSH term and keywords including (Prostatic Hyperplasia/OR Lower urinary tract symptoms) AND (holmium laser prostatectomy OR green light laser photoselective vaporization) AND (functional outcomes OR reoperation), using the ‘.mp’ modifier for keywords to broaden the results captured. A gold test set of relevant studies was used to ensure the search terms retrieved all of the gold test set. The results of the literature search were downloaded into EndNote™ X9 software (Clarivate Analytics, London, UK). Exact article duplicates were removed using the duplicate tool in Endnote™ X9 software. Subsequently, a reference review of identified articles and reviews was conducted to identify any pertinent articles. Grey literature was searched via guidelines from EAU, AUA and NICE and ongoing clinical trials through ClinicalTrials.gov, The ISRCTN registry and the World Health Organisation International Clinical Trials Registry Platform portal. Authors of trials were contacted for preliminary or unpublished results for inclusion in the review. Full search strategy and results are provided in Appendix.

### 2.3 Selection process

Following completion of the search, all identified citations were uploaded into Covidence systematic review software (Veritas Health Innovation, Melbourne, Australia) with duplicates removed. Screening for inclusion was conducted in two phases. The first phase involved screening titles and abstracts from initial search results. The second phase involved reviewing full‐text articles against the previously stated inclusion criteria (Table 2). Both phases of screening were conducted by two independent reviewers (AY and MA). In cases of disagreements that were unable to be resolved via consensus, a third reviewer (XY) adjudicated.

### 2.4 Data collection process

Data extraction was independently conducted by two reviewers (AY and XY) onto a pre‐defined extraction sheet. Data extraction was cross‐checked independently. Due to intra‐ and inter‐study variations in follow‐up times of different patient groups, data were selectively extracted for analysis in this review. For studies in which functional outcomes were measured for all patients at defined follow‐up times, data were extracted only for patients who had completed follow‐up of 5 years or greater. For studies in which patients had a median follow‐up time greater than 5 years but had no defined follow‐up times, the median follow‐up times were used. With respect to adverse events, data were extracted to include overall reoperation rate throughout the follow‐up period with specific cause for reintervention outlined where available.

### 2.5 Study risk of bias assessment

The study quality and risk of bias were assessed following the Newcastle‐Ottawa scale (NOS). Any areas of conflict between the two reviewers (AY and MA) were resolved with arbitration with a third reviewer (XY) if required.

## 3 RESULTS

**FIGURE 1:**
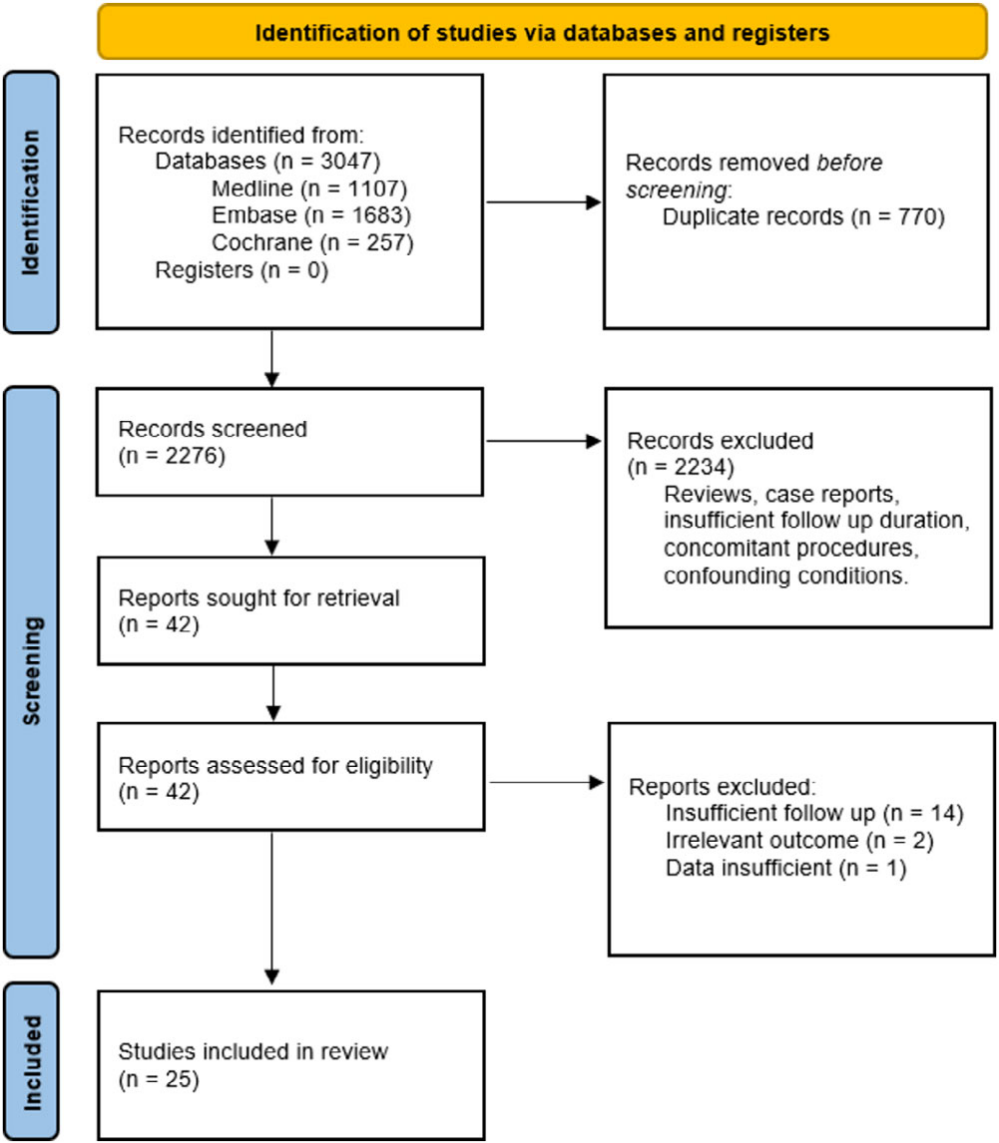
PRISMA 2020 flow diagram for study selection. PRISMA, Preferred Reporting Items for Systematic Reviews and Meta‐Analyses.

A total of 3047 articles were identified through literature search with 770 duplicates removed before screening. Of the 2276 records screened, 42 full text articles were assessed for eligibility, with 25 included for final analysis (PRISMA Flow Diagram is shown in Figure 1). Characteristics and data collected are shown in Tables 3 and 4 respectively. Study designs included two RCTs, two cohort studies, one cross‐sectional study and 20 case series. The number of participants ranged from 18 to 1216 patients. Mean follow‐up times for HoLEP studies ranged from 5 to 10.5 years for HoLEP arm and 5 to 10 years for the GLPVP arm.

**TABLE 3:** Characteristics of studies included in the systematic review.

	HoLEP	GLPVP
Studies included	10	13
Study design		
Case series	8	10
RCT	1	1
Cross‐sectional study	1	0
Cohort study	0	2
Sample size		
Sum	2608	1555
Median (IQR)	126 (83–189)	75 (28–158)
Mean (sd)	260 (368)	119 (103)
Range	38–1216	18–377
Model used		
80 W	‐	10
120 W	‐	4
180 W	‐	3
Follow‐up time (years)		
Median (IQR)	6.2 (5.1–7.6)	5.0 (4.1–5.1)
Mean (sd)	7.3 (1.9)	5.5 (1.9)
Range	5.0–10.5	5.0–10.0
Improvement in functional outcomes (mean [range])		
IPSS	74.6% (58.0%–86.4%)	60.5% (31.7%–79.1%)
QoL	66.1% (52.0%–81.6%)	61.8% (35.0%–81.0%)
Qmax	201.1% (77.8%–539%)	124.4% (46.0%–235.0%)
PVR	80.7% (53.4%–96.2%)	71.9% (32.6%–84.6%)
Adverse Events (mean [range])		
BNC	1.5% (0%–4.7%)	3.9% (1.2%–9.6%)
US	2.3% (1.2%–4.7%)	3.5% (0%–13.3%)
Residual adenoma	1.1% (0%–3.1%)	6.4% (0.7%–17.7%)
Persistent UI	7.1% (0.5%–21%)	1.2% (0%–2.1%)
Overall reoperation rate	4.1% (2.0%–6.3%)	12.6% (3.8%–33.3%)

NB: Two further (comparative) studies are in Table 4.

Abbreviations: BNC, bladder neck contracture; GLPVP, GreenLight photoselective vaporisation of prostate; HoLEP, holmium laser enucleation of prostate; IPSS, international prostate symptom score; IQR, interquartile range; PVR, post void residual; Qmax, peak flow rate; QOL, quality of life; RCT, randomised control trial; sd, standard deviation; UI, urinary incontinence; US, urethral stricture.

**TABLE 4:** Summary of all data collected for the included studies.

Author, year, study type	Technique	Sample sizerecruited /Total (%) *N* (%)	Mean/median follow‐up time (years)	Follow‐up data collection time (years)	Functional outcomes				Adverse events				
					Mean/median IPSS change	Mean/median QoL change	Mean/median Qmax change (ml/s)	Mean/median PVR change (ml)	BNC	US	Regrowth or residual adenoma	Overall reoperation rate	Persistent UI
Gilling^8^ 2008 Case series	HoLEP	38/71 (53.5%)	6.1	5.0	25.7 → 8.5 66.9% improvement	4.9 → 1.8 63.3% improvement	8.1 → 19 134.6% improvement	105.0 → 33.3 68.3% improvement	0 (0%)	1 (1.4%)	1 (1.4%)	2 (2.8%)	8 (21%)
Kuntz^9^ 2008 RCT	HoLEP	42/60 (70%)	5.0	5.0	22.1 → 3.0 86.4% improvement	N/A	3.8 → 24.3 539% improvement	280 → 10.6 96.2% improvement	1 (1.7%)	2 (3.3%)	0 (0%)	3 (5.0%)	Not evaluated after 18 months.
Krambeck^10^ 2010 Case series	HoLEP	83/1065 (7.4%)	7.0	7.0	20.3 → 5.1 74.9% improvement	N/A	8.4 → 22.7 170% improvement	N/A	16 (1.5%)	24 (2.3%)	1 (0.1%)	41 (3.8%)	6 (7.2%)
Elmansy^11^ 2011 Case series	HoLEP	89/949 (9.4%)	5.1	10.0	19 → 3.8 81.1% improvement	3.8 → 0.7 81.6% improvement	8 → 23.4 236.3% improvement	311 → 52 83.4% improvement	7 (0.8%)	15 (1.6%)	6 (0.7%)	29 (3.1%)	14 (1.5%)
Elkoushy^12^ 2015 Case series	HoLEP	1216/1216 (100%)	7.6	7.6	18.5 → 6.8 76% improvement	3.7 → 1.5 60% improvement	6.5 → 18 162% improvement	348 → 41 88% improvement	14 (1.2%)	25 (2.1%)	13 (1.1%)	52 (4.3%)	6 (0.5%)
Ibrahim^13^ 2019 Case series	HoLEP	132/1476 (8.9%)	9.1	10.0	15.9 → 6.8 58% improvement	3.1 → 1.5 52% improvement	7.2 → 17.7 145.8% improvement	204 → 43 78.9% improvement	30 (2.1%)	21 (1.4%)	21 (1.4%)	72 (4.9%)	24 (16.2%)
Enikeev^14^ 2020 Case series	HoLEP	127/347 (36.6%)	5.5	5.0	22.0 → 6.2 73% improvement	4.0 → 1.7 57.5% improvement	7.7 → 19.8 157% improvement	73.0 → 34.1 53.4% improvement	2 (1.6%)	4 (3.1%)	4 (3.1%)	10 (6.3%)	7 (5.5%)
Fallara^15^ 2021 Case series	HoLEP	125/197 (63.4%)	10.5	10.5	N/a → 5	N/a	9 → 16 77.8% improvement	82.5 → 10 87.9% improvement	6 (4.7%) combined		0 (0%)	6 (4.7%)	7 (5.7%)
Droghetti^16^ 2021 Case series	HoLEP	567/798 (71.1%)	6.3	5.0	21 → 5 76.2% improvement	3.8 → 1.0 73.7% improvement	8 → 23 187.5% improvement	100 → 10 90% improvement	8 (1.4%)	14 (2.2%)	3 (0.5%)	25 (4.4%)	24 (4.2%)
Gild^17^ 2023 Cross‐sectional study	HoLEP	189/2566 (7.3%)	4.3	7.9	19 → 4 78.9% improvement	4 → 1 75.0% improvement	Baseline 10 No data on follow‐up	Baseline 81 No data on follow‐up	2 (0.3%)	9 (1.2%)	6 (2.7%)	15 (2.0%)	17 (2.2%)
Ruszat^18^ 2008 Case series	GL PVP 80 W	27/500 (5.4%)	2.5	5.0	18.3 → 7.6 58.5% improvement	3.8 → 1.5 60.5% improvement	8.4 → 17.5 108.3% improvement	208 → 32 84.6% improvement	18 (3.6%)	22 (4.4%)	34 (6.8%)	74 (14.8%)	6 (1.2%)
Hai^19^ 2009 Case series	GL PVP 80 W	246/321 (76.6%)	5.0	5.0	24.0 → 5.0 79% improvement	4.2 → 0.8 81% improvement	8.6 → 21.1 172% improvement	170 → 28 77% improvement	3 (1.2%)	N/A	19 (7.7%)	22 (8.9%)	N/A
Elshal^20^ 2012 Case series	GL PVP 80 W (n = 91) 120 W (n = 197)	75/288 (26%)	3.3	5.0	18.1 → 6.9 61.8% improvement	3.6 → 1.3 63.9% improvement	7.6 → 16.6 118.4% improvement	256.4 → 52 79.7% improvement	10 (3.4%)	6 (2.1%)	6 (2.1%)	22 (7.6%)	N/A
Malde^21^ 2012 Case series	GL PVP 80 W	56/131 (42.7%)	5.0	5.0	22 → 9 58.4% improvement	N/A	8.0 → 13.9 72.4% improvement	321 → 76 76.5% improvement	3 (3.3%)	0 (0%)	16 (17.7%)	19 (21%)	1 (0.9%)
Elshal^22^ 2013 RCT	GL PVP 80 W	47/52 (90.3%)	5.9	7.0	18.5 → 8.1 53.8% improvement	3.6 → 1.6 55.6% improvement	6.5 → 16.5 154.6% improvement	215 → 58.5 72.8% improvement	5 (9.6%)	4 (7.7%)	4 (7.7%)	13 (25%)	N/A
Elshal^23^ 2014 Case series	GL PVP 80 W (n = 48) 120 W(n = 96)	?/144 (9%)	5.0	5.0	19 → 6 62.8% improvement	Detailed data not available. 57.2% improvement	7.5 → 19.5. 167.1% improvement	Detailed data not available. 73% improvement	9 (6.2%)	5 (3.5%)	1 (0.7%)	15 (10.4%)	3 (2.1%)
Guo^24^ 2015 Prospective cohort study	GL PVP 80 W	30/120 (25%)	5.0	5.0	19.4 → 6.2 66% improvement	3.7 → 1.3 70.6% improvement	8.3 → 15.1 81.9% improvement	119.5 → 34.5 71.1% improvement	1 (3.3%)	4 (13.3%)	5 (16.7%)	10 (33.3%)	N/A
Otsuki^25^ 2016 Case series	GL PVP 80 W	153/457 (33.7%)	5.0	5.0	21.0 → 9.03 57% improvement	5.28 → 2.03 61.6% improvement	8.76 → 17.0 94.1% improvement	107 → 25.4 76.3% improvement	7 (4.6%)	3 (1.7%)	2 (1.8%)	10 (6.5%)	N/A
Yamada^26^ 2016 Case series	GL PVP 80 W	18/1154 (1.6%)	2.9	10.0	21.0 → 10.7 49% improvement	5.0 → 2.5 50% improvement	7.4 → 7.5 No significant improvement	95 → 64 32.6% improvement	N/A	4 (0.3%)	N/A	54 (4.7%)	N/A
Park^27^ 2017 Case series	GL PVP 120 W	159/278 (57.2%)	5.0	5.0	18.9 → 12.9 31.7% improvement	4.0 → 2.6 35% improvement	10.0 → 14.6 46% improvement	80.2 → 29.3 63.5% improvement	2 (1.2%) combined		5 (3.1%)	7 (4.3%)	N/a
Ajib^28^ 2018 Case series	GL PVP 180 W	66/370 (17.8%)	4.9	5.0	26.2 → 6.5 75.2% improvement	4.7 → 1 78.7% improvement	5.5 → 18.4 235% improvement	345 → 53.7 84.4% improvement	7 (1.9%)	3 (0.8%)	4 (1.1%)	14 (3.8%)	0 (0%)
Ghobrial^29^ 2023 Cohort study	GL PVP 180 W	157/157 (100%)	5.2	5.0	25 → 9.8 61% improvement	Baseline 5 No data on follow‐up	9 → 19.7 119% improvement	Baseline 21 No data on follow‐up	2 (1.3%)	2 (1.3%)	11 (7.0%)	15 (9.6%)	3 (1.9%)
Özveren^30^ 2023 Case series	GL PVP 80 W (*n* = 72) 120 W(*n* = 198) 180 W (*n* = 107)	377/411 (85.5%)	5.1	5.1	N/a	N/a	N/a	N/a	29 (7.7%)	21 (5.6%)	18 (4.8%)	52 (13.8%)	N/a

Author, year, study type	Technique	Sample size/total recruited (%)	Mean/median follow‐up time (years)	Follow‐up data collection time	Mean/median IPSS change	Mean/median QoL change	Mean/median Qmax (mL/s) change	Mean/median PVR (mL) change	BNC	US	Regrowth or residual adenoma	Overall reoperation rates	Persistent UI
Cho^31^ 2018 Comparative case series	HoLEP (*n* = 101)	101/648 (15.6%)	5.0	5.0	18.9 → 9.8 48.15% improvement	4.1 → 2.2 46.34% improvement	11.9 → 18 51.26% improvement	64.6 → 20 69.04% improvement	1 (1.0%)	2 (2.0%)	(0) 0%	3 (3.0%)	N/A
	GL PVP 120 W (*n* = 165)	165/357 (46.2%)	5.0	5.0	19.5 → 13 33.33% improvement	4.0 → 2.6 35% improvement	11.1 → 13 17.12% improvement	82.8 → 23 72.22% improvement	2 (1.2%)	3 (1.8%)	9 (5.5%)	14 (8.5%)	N/a
					Comparison between HoLEP and PVP: Improvements in IPSS, QoL score, and Qmax were all significantly higher in HoLEP group at all points during follow‐up, except for at 36 months for Qmax. Improvement in PVR is not significantly different between HoLEP and PVP at all points during follow‐up, except for at 48 months follow‐up which showed significantly greater improvement in PVR for the HoLEP group.=								
Sun^32^ 2019 Comparative case series	HoLEP (*n* = 754)	754/1005 (75.0%)	5.0	5.0	19.2 → 9 47.9% improvement	4.2 → 2.4 43.1% improvement	10.3 → 18 74.8% improvement	67.2 → 17 74.4% improvement	2 (0.5%)	18 (2.4%)	0 (0%)	20 (2.7%)	N/a
	GL PVP 120 W (*n* = 439)	439/564 (77.8%)	5.0	5.0	20.6 → 13 36.89% improvement	4.3 → 1.9 55.3% improvement	10.6 → 11 3.77% improvement	83.2 → 25 70.0% improvement	1 (0.2%)	2 (0.5%)	12 (2.7%)	15 (3.4%)	N/a
					Comparison between HoLEP and PVP: Improvement in all functional outcomes were significantly higher in HoLEP group at 60 months follow‐up, except for mean PVR.								

Abbreviations: BNC, bladder neck contracture; GLPVP, GreenLight photoselective vaporisation of prostate; HoLEP, holmium laser enucleation of prostate; IPSS, international prostate symptom score; PVR, post void residual; Qmax, peak flow rate; QOL, quality of life; RCT, randomised control trial; UI, urinary incontinence; US, urethral stricture.

Twenty‐three studies provided functional outcomes, adverse event incidence and reoperation rates on either HoLEP or GLPVP, while two studies compared HoLEP and GreenLight PVP to each other. At baseline, the mean IPSS ranged from 15.9 to 25.7, mean QoL ranged from 3.1 to 5.3, mean Qmax ranged from 3.8 to 11.9 ml/s and mean PVR ranged from 64.6 to 348 mL.

For the HoLEP arm, one RCT, one cross‐sectional study and eight case series were included. Four studies reported findings at 5 years, three between at 7 and 7.9 years and three at 10 to 10.5 years. The mean follow‐up time for all HoLEP patients was 7.3 years. The mean improvement of several functional outcomes ranged from IPSS 58%–86.4%, QoL 52%–81.6%, Qmax 77.8%–539% and PVR 53.4%–96.2%. Adverse events ranged from bladder neck contracture (BNC) 0%–4.7%, urethral stricture (US) 1.2%–4.7%, regrowth or residual adenoma 0%–3.1%, persistent urinary incontinence 0.5%–21% and overall reoperation rate 2%–6.3%.

For the GLPVP arm, one RCT, two cohort studies and 10 case series were included. The majority of studies reported follow‐up data at 5 years (*n* = 11), whilst one study reported at 7 years and another at 10 years. The mean follow‐up time for all GLPVP patients was 5.5 years. The mean improvement of several functional outcomes ranged from IPSS 31.7%–79%, QoL 35%–81%, Qmax 46%–253% and PVR 32.6%–84.6%. Adverse events ranged from BNC 1.2%–9.6%, US 0%–13.3%, regrowth or residual adenoma 0.7%–17.7%, persistent urinary incontinence 0%–2.1% and overall reoperation rate 3.8%–33.3%.

Two comparative case series had follow‐up data of 5 years. HoLEP patients had improvement in the mean range IPSS 47.9%–48.15%, QoL 43.1%–46.34%, Qmax 51.26%–74.8% and PVR 69.04%–74.4%. Adverse events ranged from BNC 0.5%–1%, US 2%–2.4%, regrowth or residual adenoma 0% and overall reoperation 2.7%–3%. Whilst GLPVP patients had improvement in the mean range IPSS 33.33%–36.89%, QoL 35%–55.3%, Qmax 3.77%–17.12% and PVR 70%–72.22%. Adverse events ranged from BNC 0.2%–1.2%, US 0.5%–1.8%, regrowth or residual adenoma 2.7%–5.5% and overall reoperation 3.4%–8.5%. Table 5 demonstrates risk of bias and quality assessment of non‐randomised studies with a Newcastle–Ottawa score ranging from 5 to 9 for the included studies.

**TABLE 5:** Newcastle–Ottawa scale for the risk of bias and quality assessment of non‐randomised studies.

Author, study number	Year	Selection				Comparability	Exposure			Total score
Case controls ‐‐>		Adequate definition of patient cases	Representativeness of patient cases	Selection of controls	Definition of controls	Control for important or additional factors	Ascertainment of exposure	Same method of ascertainment for participants	Nonresponse rate	
Cohort studies ‐‐>		Representiveness of the exposed cohort	Selection of nonexposed cohort	Ascertainment of exposure	Demonstration that outcome of interest was not present at the start of study	Comparability of cohorts on the basis of the design or analysis	Assessment of outcome	Was follow‐up long enough for outcomes to occur	Adequacy of follow‐up of cohorts	
Gilling 12008Case series	HoLEP	⋆	⋆	⋆	⋆	⋆	⋆	⋆		**7**
Kuntz 22008RCT	HoLEP	⋆	⋆	⋆	⋆	⋆⋆	⋆	⋆	⋆	**9**
Krambeck 32010Case series	HoLEP	⋆		⋆	⋆	⋆		⋆		**5**
Elmansy 42011Case series	HoLEP	⋆		⋆	⋆	⋆	⋆	⋆	⋆	**7**
Elkoushy 52015Case series	HoLEP	⋆		⋆	⋆	⋆	⋆	⋆		**6**
Ibrahim 62019Case series	HoLEP	⋆		⋆	⋆	⋆	⋆	⋆		**6**
Enikeev 72020Case series	HoLEP	⋆		⋆	⋆	⋆	⋆	⋆		**7**
Fallara 82021Case series	HoLEP	⋆		⋆	⋆	⋆⋆	⋆	⋆	⋆	**8**
Droghetti 92021Case series	HoLEP	⋆		⋆	⋆	⋆⋆	⋆	⋆	⋆	**8**
Gild 102023 Cross‐sectional study	HoLEP	⋆		⋆	⋆	⋆⋆	⋆	⋆		**7**
Ruszat 112008Case series	GL PVP 80 W	⋆		⋆	⋆	⋆	⋆	⋆	⋆	**7**
Hai 122009Case series	GL PVP 80 W	⋆		⋆	⋆	⋆	⋆	⋆	⋆	**7**
Elshal 132012Case series	GL PVP 80 W (*n* = 91) 120 W (*n* = 197)	⋆		⋆	⋆	⋆⋆	⋆	⋆		**7**
Malde 142012Case series	GL PVP 80 W	⋆		⋆		⋆	⋆	⋆		**5**
Elshal 152013RCT	GL PVP 80 W	⋆	⋆	⋆	⋆	⋆⋆	⋆	⋆	⋆	**9**
Elshal 162014Case series	GL PVP 80 W (*n* = 48)120 W (*n* = 96)	⋆		⋆	⋆	⋆⋆	⋆	⋆		**7**
Guo 172015Prospective cohort study	GL PVP 80 W	⋆		⋆	⋆	⋆	⋆	⋆		**6**
Otsuki 182016Case series	GL PVP 80 W	⋆	⋆	⋆	⋆	⋆	⋆	⋆		**7**
Yamada 192016Case series	GL PVP 80 W	⋆		⋆	⋆	⋆	⋆	⋆		**6**
Park 202017Case series	GL PVP 120 W	⋆	⋆	⋆	⋆	⋆⋆	⋆	⋆		**8**
Ajib 212018Case series	GL PVP 180 W	⋆	⋆	⋆		⋆⋆	⋆	⋆	⋆	**8**
Ghobrial 222023 Cohort study	GL PVP 180 W	⋆		⋆	⋆	⋆⋆	⋆	⋆		**7**
Özveren 232023 Case series	GL PVP 80 W (*n* = 72) 120 W (*n* = 198) 180 W (*n* = 107)	⋆	⋆	⋆	⋆	⋆⋆	⋆	⋆	⋆	**9**
Cho 242018Comparative case series	HoLEP (*n* = 101) GL PVP 120 W (*n* = 165)	⋆	⋆	⋆	⋆	⋆	⋆	⋆	⋆	**8**
Sun 252019Comparative case series	HoLEP (*n* = 754) GL PVP 120 W (*n* = 439)	⋆	⋆	⋆	⋆	⋆⋆	⋆	⋆		**8**

Abbreviations: GLPVP, GreenLight photoselective vaporisation of prostate; HoLEP, holmium laser enucleation of prostate.

## 4 DISCUSSION

### 4.1 Comparing functional outcomes

This review found compelling evidence in support of the long‐term efficacy and durability of HoLEP at follow‐up times from 5 to 10 years. All studies providing long‐term data after HoLEP demonstrated significant improvements for both subjective (IPSS, QoL) and objective (PVR, Qmax) functional outcomes until final follow‐up. In comparison, GLPVP demonstrated functional improvements to a lesser degree.

For subjective functional outcomes, HoLEP saw an average improvement of 74.6% and 66.1% for IPSS and QoL respectively, whereas GLPVP demonstrated improvements of 60.5% and 61.8%. The highest mean IPSS at the end of follow‐up period for HoLEP was 8.5 in a case series by Gilling et al (2008),^8^ although notably, this was one of the earliest studies. All subsequent nine out of 10 studies demonstrated IPSS scores less than 8 which corresponds with ‘mild’ symptoms only. This is likely attributable to improvements in surgeons' experience and technique as the technology was being adopted. In comparison, the highest mean IPSS for GLPVP was 12.9 in a case series by Park et al. (2017),^27^ with another six out of 13 studies reporting IPSS scores greater than 8 following GLPVP, corresponding with ‘moderate’ symptoms at 5 years.^20^, ^25^, ^26^, ^27^, ^29^, ^33^

QoL scores were generally better for the HoLEP group, with all seven studies reporting these data showing scores less than 2, corresponding to ‘pleased’ or ‘mostly satisfied’. In comparison, three of the 10 studies available for GLPVP showed inferior QoL scores greater than 2,^25^, ^26^, ^27^ corresponding to ‘mixed satisfaction’.

For objective functional outcomes, HoLEP also outperformed GLPVP, with an average improvement of 201% for Qmax and 80% for PVR, compared with 124% and 72% for GLPVP. Although a fairly crude measure, the best mean Qmax of 24.3 mL/s was reported by Kuntz et al. (2008)^9^ 5 years following HoLEP, whereas the best mean Qmax 5 years following GLPVP was 21.1 mL/s.^19^ The superiority of HoLEP is even more pronounced at 10 years, with Qmax ranging from 16 to 23.4 mL/s in the three studies available,^11^, ^13^, ^15^ whereas the only study reporting data at 10 years for GLPVP by Yamada et al.^26^ saw virtually no change in Qmax, from a baseline of 7.4 to 7.5 mL/s at final follow‐up. However, this study suffered from extreme patient attrition, with only 1.8% of the initial cohort available for analysis at 10 years. Similar findings were seen when comparing PVR, with HoLEP showing a greater and sustained improvement.

### 4.2 Comparing adverse events and reoperation rates

When comparing reoperation rates between techniques, HoLEP demonstrated superior outcomes from the long‐term literature available. The mean overall reoperation rate was 4.1% for HoLEP, compared with 12.6% for GLPVP. HoLEP studies also had a longer follow‐up time compared with GLPVP, emphasising its durability in the long‐term.

Trends of revision or reoperation rates at 5 years for GLPVP from the early 2000s have reduced from 11.6% to 8.9% to 6.1% with the introduction of 180 W lithium triborate laser and established use of the technique.^19^, ^26^, ^34^ Additionally, these rates are within the identified range of 3.8% to 33.3% and add weight to the superior rate identified for HoLEP.

The overall reoperation rate following HoLEP ranged from 2.0% to 6.3%, with only one study by Enikeev et al. (2020)^14^ reporting a rate of greater than 5%. US was the most prevalent adverse event requiring reoperation with a mean prevalence of 2.3%, followed by bladder neck contracture (1.5%) and residual adenoma (1.1%). In contrast, the most prevalent complication following GLPVP was residual adenoma (6.4%), followed by BNC (3.9%) and US (3.5%).

Overall reoperation following GLPVP ranged from 3.8% to 33.3%, with three studies reporting rates of above 20% at 21%, 25% and 33% respectively.^22^, ^24^, ^33^ However, each of these studies did have smaller than average sample sizes (30–56) and used only the 80‐W model. In one of these studies, surgeons were at the beginning of their learning curve,^21^ and in another study, reoperation was reported to be significantly associated with the use of a larger 26 F cystoscope during operation, which was later replaced by a smaller 22 F cystoscope.^24^ Notably, the two studies reporting long‐term data for the 180‐W model reported lower reoperation rates at 3.8% and 9.6% respectively.^28^, ^29^ When looking at persistent urinary incontinence, HoLEP had a mean of 7.1% (0.5%–21%) compared with GLPVP of 1.2% (0%–2.1%) (Table 3). This is in line with previous evidence, albeit more specifically to stress urinary incontinence, identified on urinary leak tests by Barnard et al.^35^ demonstrating 22% incidence post HoLEP (n = 10).

### 4.3 Comparing models and study designs

The currently available long‐term data are weighted towards the older GLPVP 80‐W model (10 studies) with less available for the later models, 120 W (four studies) and 180 W (three studies). Ruszat et al.^18^ reported higher retreatment rates in patients with larger prostate sizes than smaller prostate sizes and quoted adequate energy delivery to be an important role in sufficient ablation and reducing reoperation rates due to adenoma regrowth. Although the data collected from different GLPVP models are generally comparable, a case series by Ajib et al.^28^ utilising the latest 180‐W model showed superior improvement across all functional outcomes and a lower reoperation rate (3.8%) at 5‐year follow‐up. However, subsequent studies using the same 180‐W model reported rates of 9.6% and 13.8%,^29^, ^30^ which are higher than some 80 W studies, and all HoLEP studies examined. Therefore, these findings contradict the above theory by Ruszat et al.,^18^ as higher power GLPVP does not appear to consistently improve reoperation rates.

Follow‐up times were also longer in the HoLEP studies compared with GLPVP. Beyond 10 years, there were three studies with HoLEP data available, whilst GLPVP only had one study. HoLEP studies also had greater sample sizes, with a mean of 260 participants compared with 119 for GLPVP. The overall pooled sample size was 2608 for HoLEP and 1555 for GLPVP. Overall, the longer follow‐up times and greater sample sizes suggest the HoLEP data to be robust and reflective of real‐world outcomes.

### 4.4 Comparative studies

Two comparative studies comparing HoLEP and GLPVP reported significant improvements from baseline in all subjective and objective functional outcomes at the latest follow‐up for both arms. Sun et al.^32^ reported all functional parameters for both arms are significantly improved compared with baseline, with the exception of Qmax for the GLPVP arm. Comparing HoLEP to GLPVP, the HoLEP arm had significantly higher improvements in all functional parameters at 60‐month follow‐up except QoL. Cho et al.^31^ reported similar findings, with the HoLEP arm demonstrating significantly greater improvement in IPSS, QoL and Qmax, but not PVR.

Reoperation rates were again lower for the HoLEP arms in both comparative studies, despite both studies using only the 120‐W GLPVP model (3.0 vs 8.5% and 2.7 vs 3.4%). These reoperation rates are consistent with previous published studies that found an overall higher reoperation rate due to residual adenoma following GLPVP.

### 4.5 Limitations

Although HoLEP and GLPVP are commonly compared, this study acknowledges that the patient demographic of choice may differ between the two groups (large prostates >80 mL vs comorbid and anticoagulated men, respectively), which limits direct comparison (i.e. apples vs oranges). Though with the advent of advancing surgical technologies such as laser energy, comparison of similar modalities, albeit potentially different indications, remains important. Additionally, there is a profound lack of comparative studies for HoLEP and GLPVP, with only two studies reporting data beyond 5 years available for this review. Most studies available in the literature are either focused on a single procedure or are studies that compare HoLEP or GLPVP to another technique such as TURP. The lack of RCTs directly comparing HoLEP to GLPVP limits our ability to randomise baseline characteristics such as functional parameters, comorbidities, prostate volumes and anticoagulation status between HoLEP and GLPVP arms. As a result, there is intra‐ and inter‐study heterogeneity when comparting patient populations that may impact patient trajectory postoperatively.

Furthermore, an attrition bias may contribute to interpretation of the results given only three studies have attrition rates less than 10%.^12^, ^22^, ^29^ The worst performer was Yamada et al (2016) who had 1.8% of patients available for 10‐year data collection and a median retention rate of 33.7%.^26^ Unfortunately, attrition bias is a common limitation for studies with long follow‐up times which may be magnified in BPH studies due to an older population. Interestingly, comorbid patients, those with late adverse events and eventual reoperations, tend to have longer follow‐up times which may also lead to selection bias that provides insight into a real‐world patient population.

The surgeon learning curve may also impact the validity of results with underreporting of surgeon experience. Clearly, surgeons with less experience are more likely to have complications compared with those who have mastered the technique. It is recommended for HoLEP in particular to have a minimum of 20–30 cases and a fellowship accreditation to ensure safe usage and familiarity of the device before operating independently.^36^ Similarly, for GLPVP, we note that Malde et al. (2012) reported a reoperation rate of 21% for GLPVP with three surgeons having a combined experience of 38 cases, suggesting the importance of surgeon experience to the outcomes of interest for intervention studies.^21^

Another important limitation for the GLPVP arm includes the higher weighting on the 80‐W model. Given the recent introduction of the 120‐ and 180‐W models, data maturation is required for confirmation of superiority. At 10‐year follow‐up, only one study was available for GLPVP 80 W^26^ and none for the 120‐W/180‐W models, in contrast with three studies available for HoLEP. This emphasises the need for further studies into the longer‐term outcomes of the two techniques.

## 5 CONCLUSIONS

Overall, our findings are consistent with previously published reviews and supports the long‐term durability and efficacy of HoLEP for BPH. GLPVP, on the other hand, reaches the 5‐year efficacy of treatment without clear evidence at 10 years. However, with data maturation and progressive use of the 120‐W and 180‐W models, we may yet see robust evidence in the coming years. HoLEP appears to provide greater functional improvements at 5‐year follow‐up and lower adverse outcomes, including reoperation rate, when compared with the GLPVP 80‐W model. However, results are limited by small sample sizes, significant patient attrition due to long‐term follow‐up requirements and lack of data for the 120‐W/180‐W models. The authors recommend further large cohort prospective studies comparing HoLEP to GLPVP 180 W for development of best surgical practice.

## AUTHOR CONTRIBUTION

AY, MA and XY: writing—original draft preparation, writing—reviewing and editing, data curation; DB and LMW: writing—reviewing and editing; KS: conceptualisation, methodology, supervision.

## CONFLICT OF INTEREST STATEMENT

The authors declare no conflicts of interest.

## APPENDIX A

### Full Search Strategy

Embase <1974 to 2023 November 03>.

1. prostate hypertrophy/42271
2. Benign prostatic hyperplasia*.mp. 23192
3. (Lower urinary tract symptom* or LUTS).mp.25815
4. 1 or 2 or 3 62432
5. (holmium laser or holmium powered laser enucleation of prostate or holmium laser prostatectomy or HoLEP or holmium or holmium enucleation).mp.10258
6. (green light or green light laser or green light laser prostatectomy or photoselective vaporization or PVP).mp.18214
7. 5 or 6 28215
8. (reoperat* or repeat surgery or return to theatre or longevity or follow‐up or result* or durab* or long‐term or lasting).mp.18550744
9. ((Function* or function* outcome* or incontinen* or IPSS or International prostatic symptoms score or QoL or quality of life) and (post‐operat* or perioperat* or after surgery or post‐surg*)).mp.164925
10. 8 or 9 18576000
11. 4 and 7 and 10 2568
12. exp animals/not humans/11683135
13. 11 not 12 1845
14. limit 13 to english language 1740
15. limit 14 to yr="2004‐2024" 1683

Ovid MEDLINE(R) ALL <1946 to November 03, 2023>

13 Prostatic Hyperplasia/ 24251

14 Benign prostatic hyperplasia*.mp. 16267

15 (Lower urinary tract symptom* or LUTS).mp. 12490

16 13 or 14 or 15 36795

17 (holmium laser or holmium powered laser enucleation of prostate or holmium laser prostatectomy or HoLEP or holmium or holmium enucleation).mp. 4074

18 (green light or green light laser or green light laser prostatectomy or photoselective vaporization or PVP).mp. 14312

19 17 or 18 18273

20 (reoperat* or repeat surgery or return to theatre or longevity or follow‐up or result* or durab* or long‐term or lasting).mp. 13762161

21 ((Function* or function* outcome* or incontinen* or IPSS or International prostatic symptoms score or QoL or quality of life) and (post‐operat* or perioperat* or after surgery or post‐surg*)).mp. 95147

22 20 or 21 13778910

23 16 and 19 and 22 1306

24 exp animals/not humans/ 5167250

25 23 not 24 1299

26 limit 25 to english language 1149

27 limit 26 to yr="2004‐2024" 1107

Cochrane ‐ Date Run: 06/11/2023 14:20:57

ID Search Hits

#1 [mh ^"prostate hypertrophy"] 0

#2 ("Benign prostatic" NEXT hyperplasia*):ti,ab,kw 2882

#3 (("Lower urinary tract" NEXT symptom*):ti,ab,kw OR LUTS:ti,ab,kw) 2942

#4 #1 OR #2 OR #3 4666

#5 ("holmium laser":ti,ab,kw OR "holmium powered laser enucleation of prostate":ti,ab,kw OR "holmium laser prostatectomy":ti,ab,kw OR HoLEP:ti,ab,kw OR holmium:ti,ab,kw OR "holmium enucleation":ti,ab,kw) 771

#6 ("green light":ti,ab,kw OR "green light laser":ti,ab,kw OR "green light laser prostatectomy":ti,ab,kw OR "photoselective vaporization":ti,ab,kw OR PVP:ti,ab,kw) 829

#7 #5 OR #6 1568

#8 (reoperat*:ti,ab,kw OR "repeat surgery":ti,ab,kw OR "return to theatre":ti,ab,kw OR longevity:ti,ab,kw OR follow‐up:ti,ab,kw OR result*:ti,ab,kw OR durab*:ti,ab,kw OR long‐term:ti,ab,kw OR lasting:ti,ab,kw) 1135891

#9 ((Function*:ti,ab,kw OR (function* NEXT outcome*):ti,ab,kw OR incontinen*:ti,ab,kw OR IPSS:ti,ab,kw OR "International prostatic symptoms score":ti,ab,kw OR QoL:ti,ab,kw OR "quality of life":ti,ab,kw) AND (post‐operat*:ti,ab,kw OR perioperat*:ti,ab,kw OR "after surgery":ti,ab,kw OR post‐surg*:ti,ab,kw)) 24385

#10 #8 OR #9 1142481

#11 #4 AND #7 AND #10 257

#12 [mh animals] NOT [mh ^humans] 2951

#13 #11 NOT #12 257
